# A Case of Inversio Uteri Totalis Post Partum—Acute Anemia—Intravenous Transfusion of Common Salt Solution—Recovery

**Published:** 1889-10

**Authors:** A. Bergstrand, L. G. Sellstedt


### A CASE OF INVERSIO UTERI TOTALIS POST PARTUM. ACUTE ANEMIA—INTRAVENOUS TRANSFUSION OF COMMON SALT SOLUTION—RECOVERY.

Communicated by Dr. A. BERGSTRAND.

Translated from *Hygiea*, by Mr. L. G. Sellstedt.

The patient, a married woman, 32 years of age, *primipara*, had been delivered by a midwife. The delivery had not exhibited anything unusual, and the midwife denies having sought to loosen the afterbirth by pulling on the navel-string or other manipulations. While she was washing the child, she discovered that the blood was streaming from the woman, and, on investigation, the uterus was found lying like a great swelling in the vulva, while all the time a violent bleeding took place. Frightened and confused, she sent for two physicians, who immediately made their appearance. They ordered that the patient be at once brought to the hospital, which was near at hand.

How, and under what circumstances, she was transported is unknown to me, but when I instantly made my appearance she was without pulse, and utterly collapsed. She lay with closed eyes, but was able to open them, although the look was staring and lifeless. Consequently, she was not insensible, although very nearly so. The bleeding had now ceased. In the vulva lay a swelling, doughy and velvety to the touch, which proved to be the crowded and fully inverted uterus. The outward-turned mucous membrane was blood-red. How long time had passed since the afterbirth had gone off, cannot with certainty be determined; probably some hours, however. Copious ether injections in both arms were made without delay, and the cold legs were quickly swathed. After a hasty rinsing with quite warm solution of boracic acid, an attempt at reposition was made with well-disinfected hands. This failed, in spite of energetical attempts. These attempts at reposition were made by carrying the left hand above the symphysis into the funnel-shaped hollow of the uterus; with the other, an endeavor was, at the same time, made to carry it up towards the projecting fundus uteri. For fear that too strong pressure might rupture the wall of the uterus, and especially as the patient, at the attempts of reposition, awoke out of her coma and screamed out, it was determined to relax the tissues by means of a mild narcotic. As chloroform here could not, without danger, be resorted to, hypnotic suggestions and strokings were tried. These, though they did not result in full insensibility, acted so that the reposition succeeded. The hand now lay inside of the uterus, and was tolerably firmly covered by it. After it was taken out, an irrigation in the uterus was made with warm 3 p. c. solution of carbolic acid, immediately followed by a solution of boracic acid.

All hemorrhage was now arrested, but the patient was utterly prostrated, and seemed to be dying. No pulse could be felt, and the respiration was weak and superficial. Renewed ether injections were made, and when these had no visible effect, it was determined to make a transfusion with a solution of common salt. A provisional apparatus was arranged for transfusion; a common glass dropper, such as is used for dropping fluids into the eye, was fastened to a drainage-tube, the other end of which was strained over the glass tube of an irrigator with a rubber tube. The liquid which filled the irrigator (the transfusion liquid) was happily found at hand, and consisted of six grams of chlorate of soda and three grams of hydrate of soda, dissolved in a liter of distilled water, which was kept at a temperature of 38° C. The median vein was isolated, exposed, and opened sufficiently to admit the glass canula, after which by a pretty hard pressing of the irrigator, slowly, about half a liter was injected. When the liquid in the irrigator no longer sank, the operation was discontinued.

The result was surprising and obvious. The pulse began to be felt, and the face acquired life. The patient opened her eyes. If it was the transfusion alone which saved the patient, or in what degree the other injections had contributed, I leave undetermined. That the transfusion brought about a manifest action, cannot be doubted. Under the continued endeavors, by means of stimulants, to raise the sunken powers, the patient gained strength, but the danger was not yet over. Already, after a couple of days, a septic endometritis, with high fever, broke out. This was treated with thorough intra-uterine washes, morning and evening, with a 3 p. c. carbolic solution, and directly afterwards with solution of boracic acid, for fear of carbolic poisoning. The temperature began to sink, and the puerperal fever was conquered. The puerpery afterwards ran its course normally. She gradually recovered her strength, while keeping her bed for a somewhat prolonged period. After several months, she reported at reception hour with a blooming appearance.

The question of the intravenous and the subcutaneous transfusion has been treated by Ziemssen in an interesting paper in his Klinische Vorträge (1887, No. 3). Owing to the many dangers and difficulties accompanying the venous infusion, he makes himself an earnest advocate for the subcutaneous, which he has used with defibrinated blood on various occasions, as well in anemias as in poisonings. The technique of the latter is simpler. Ziemssen, however, holds that a bleeding which threatens mortal results requires a transfusion of defibrinated blood. The infusion of salt may for a time delay, but cannot prevent death.

He thinks the subcutaneous common salt infusion particularly adapted to puerperal hemorrhages, where time and place often exclude the various more complicated processes. Here, all that is needed is a syringe (best, a horse morphine syringe), chloride of soda powder, about seven grams to the liter of boiled water. It is well, after the subcutaneous injection, to use massage, to spread the liquid. The syringe ought to hold about thrirty grams. Ziemssen's method was unknown to me when I met the above mentioned case; but if it should be shown that the subcutaneous injection works as well as the transvenous infusion, the former ought to be preferred, in view of its more simple technique.

